# Defining Nutritional and Functional Niches of Legumes: A Call for Clarity to Distinguish a Future Role for Pulses in the Dietary Guidelines for Americans

**DOI:** 10.3390/nu13041100

**Published:** 2021-03-27

**Authors:** Chelsea Didinger, Henry J. Thompson

**Affiliations:** 1 Department of Food Science and Human Nutrition, Colorado State University, Fort Collins, CO 80523, USA; chelsea.didinger@colostate.edu; 2 Cancer Prevention Laboratory, Colorado State University, Fort Collins, CO 80523, USA

**Keywords:** legumes, pulses, dietary fiber, Dietary Guidelines for Americans, oilseed legumes, nutrition, beans, peas, lentils

## Abstract

Legume food crops can contribute to the solution of diet-related public health challenges. The rich diversity of the botanical family Fabaceae (Leguminosae) allows legumes to fill numerous nutritional niches. Pulses (i.e., a subgroup of legumes including chickpeas, cowpeas, dry beans, dry peas, and lentils) are a nutrient-dense food that could play a key role in eliminating the dramatic underconsumption of dietary fiber and potassium, two dietary components of public health concern, all while maintaining a caloric intake that promotes a healthy weight status. However, incorrect use of terminology—in the commercial and scientific literature as well as in publications and materials prepared for the consuming public—creates confusion and represents a barrier to dissemination of clear dietary guideline messaging. The use of accurate terminology and a simple classification scheme can promote public health through differentiation among types of legumes, better informing the development and implementation of nutritional policies and allowing health care professionals and the public to capitalize on the health benefits associated with different legumes. Although inconsistent grouping of legumes exists across countries, the recently released 2020–2025 Dietary Guidelines for Americans (DGA) were chosen to illustrate potential challenges faced and areas for clarification. In the 2020–2025 DGA, pulses are included in two food groups: the protein food group and ‘beans, peas, lentils’ vegetable subgroup. To evaluate the potential of pulses to contribute to intake of key dietary components within calorie recommendations, we compared 100 kilocalorie edible portions of pulses versus other foods. These comparisons demonstrate the unique nutritional profile of pulses and the opportunity afforded by this type of legume to address public health concerns, which can be greatly advanced by reducing confusion through global harmonization of terminology.

## 1. Introduction

There are many edible legumes, at least 50, that have been identified at the level of genus-species in the botanical family Fabaceae (Leguminosae) and that are consumed by various populations around the world [1]. Advocacy for members of the Fabaceae food grouping—particularly at the levels of genus, species, and cultivar within species—has contributed to artificial distinctions that have been used to focus attention on specific entities (e.g., dry beans versus pulses when in fact dry beans are a type of pulse). Over an extended period, this practice has contributed to a confused body of terminology. To begin to rectify this situation, we adopted the following approach: (1) use an internationally accepted classification system from the Food and Agriculture Organization (FAO) as a basis for terminology [2]; (2) concentrate on predominant legume crops consumed by the global population as a whole [3]; and (3) use the United States as an example to illustrate the value of accurate and consistent use of terminology [4]. There is a strong rationale to encourage consumption of whole foods, which are positively associated with numerous health benefits, such as weight maintenance and a healthy gut microbiome [5,6]. Thus, we focus on legumes as whole foods, not as the ingredients into which they can be fractionated (e.g., pea protein powder).

## 2. Defining ‘Legume’

Although the words legume, bean, and pulse are often used interchangeably, there are clear distinctions among them. In resources from the International Year of Pulses (2016), FAO states:

Pulses are annual leguminous crops yielding between one and 12 grains or seeds of variable size, shape and color within a pod, used for both food and feed. The term ‘pulses’ is limited to crops harvested solely for dry grain, thereby excluding crops harvested green for food, which are classified as vegetable crops, as well as those crops used mainly for oil extraction and leguminous crops that are used exclusively for sowing purposes [2].

Herein, FAO clarifies three main points: (1) pulses (e.g., chickpeas, cowpeas, dry beans, dry peas, and lentils) are a subgroup of legume; (2) the developmental stage at which legumes are harvested can impact classification (i.e., vegetable versus pulse); and (3) oilseed legumes are a separate category. However, even common, reputable sources like national dietary guidelines do not always have definitions that align with that of FAO, creating a potential source of confusion for professionals and the public alike.

### Classification Scheme

**Figure 1:**
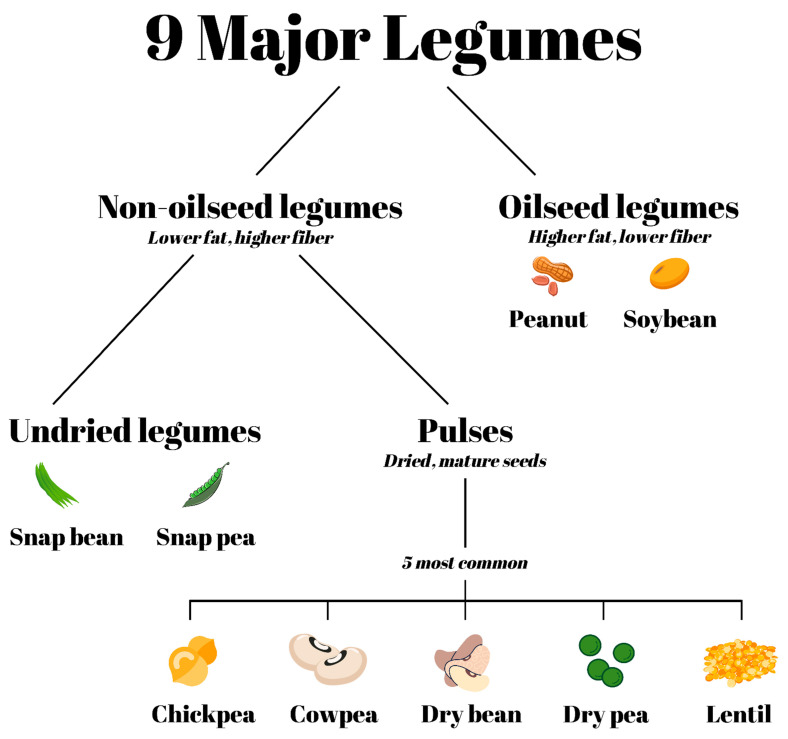
Commonly consumed types of legumes. After separating legumes into oilseed and non-oilseed legumes, non-oilseed legumes can be further divided into two categories: undried legumes and pulses. Pulses are the dried, edible seeds of grain legumes that are then cooked before being consumed.

Broadly, from the vantage point of production or consumption data, there are two categories of legumes that are eaten by people: oilseed legumes and non-oilseed legumes. Whereas oilseed legumes are higher in lipid, non-oilseed legumes are lower in fat and richer in fiber, per 100 kilocalorie (kcal) edible portion. The next level of distinction is within non-oilseed legumes and relates to whether they are harvested and consumed before or after the seeds within the pod have dried: (1) pulses are the dried, mature seeds of the pods and are sometimes called grain legumes; and (2) undried legumes are harvested before drying (i.e., green) and may be consumed with or without their pods, as is often the case for sugar snap peas and green peas, respectively (Figure 1) [3]. Overall, from a nutrient composition perspective, the profile of 100 kcal edible portions of undried legumes and pulses is similar, with comparable amounts of dietary fiber and protein. For example, green pea is the physiologically mature, undried seed with very similar nutrient composition to dry pea. However, because pulses are dried seeds, they: (1) have a longer shelf-life than their undried counterparts (unless the undried legumes are preserved through processes such as canning or freezing); and (2) should be heat treated (e.g., soaked and boiled) before eating to prevent or reduce any negative effects of so-called ‘anti-nutrients’ like lectins.

The world primarily consumes nine types of legume crops as whole foods: five of these are pulses (chickpeas, cowpeas, dry beans, dry peas, and lentils) and two are undried legumes (snap beans and snap peas) (Figure 1). In addition to these seven major non-oilseed legume crop types, there are two oilseed legumes consumed when the physiologically mature seed is harvested (peanuts and soybeans).

## 3. Are All Legumes Created Equal?

The nutritional profile of legumes can vary substantially and differentiating between the types of legumes is essential to inform clear dietary messaging to benefit public health. Currently, chronic disease causes about 70% of mortalities worldwide [7]. Yet, much of chronic disease could be prevented by reversing major risk factors, of which unhealthy diet is paramount. Overconsumption of calories and underconsumption of key beneficial dietary components like dietary fiber correlate with the dramatic increases in overweight/obesity and associated chronic diseases [8,9], whereas fiber intake positively correlates with healthy weight maintenance and lower risk for chronic diseases such as certain types of cancer (e.g., colorectal cancer) [10]. However, less than 90% of Americans consume adequate amounts of dietary fiber (defined by the Dietary Guidelines for Americans (DGA) as 14 g fiber per 1000 kcal consumed) and many achieve only 50% of the recommended intake [4]. Dietary fiber and potassium are classified as two of only four main dietary components of public health concern in the DGA. Importantly, pulses are a rich source of both and could readily remediate inadequate intake. Ultimately, delineating distinctions among the different types of legumes can help inform development of important resources and policies that impact public health and eating patterns, such as the DGA, which inform all national feeding programs, like the National School Lunch Program.

To draw comparisons across all food groups and evaluate the potential of pulses to contribute to adequate nutrient intake within calorie recommendations, we compared 100 kcal edible portions. Notably, unlike serving size measurements that vary between food groups in the DGA (e.g., cup measurements in the vegetable group and ounces for protein foods), 100 kcal portions facilitate comparisons across food groups and are thus used throughout this paper. Information in the following tables is from the United States Department of Agriculture FoodData Central [11]. Entries at the top of the search results were selected that: (1) were prepared in similar ways to allow for valid comparisons (e.g., cooked and prepared without added oil, which would falsely represent lipid content); and (2) had minimal or no missing values.

The three main categories of legumes (i.e., oilseed legumes and the two groups that comprise non-oilseed legumes, pulses and undried legumes) have different lipid and fiber composition (Table 1). For example, 100 kcal of cooked dry peas provides over 7.0 g of dietary fiber. This contrasts starkly with 100 kcal of unroasted peanuts, which provides nearly five times less dietary fiber and 30 times more fat. Compared to pulses, 100 kcal of boiled soybeans has more protein but also higher lipid and lower fiber content. Edamame and pulses have similar protein amounts on a 100 kcal basis, but again pulses are lower in fat and have approximately double the fiber, depending on the type of pulse. Clearly, the low lipid content and high, approximately 1-to-1 protein-to-fiber ratio differentiates pulses from their oilseed legume counter-parts. Furthermore, examination of the nutritional profile of non-oilseed legumes (i.e., undried legumes and pulses, like green pea versus dry pea) reveals strong overlap. Similar to pulses, a 100 kcal portion of snap beans or snap peas is low in fat and rich in dietary fiber. However, pulses have the added benefits of storing well for long periods of time and delivering the same amount of dietary fiber in a smaller portion size (e.g., ½-cup cooked dry peas versus 2.5-cups sugar snap peas) and often at a more accessible price point. Accordingly, pulses are particularly well-positioned to reverse the dramatic underconsumption of dietary fiber by much of the population, regardless of socioeconomic class. These are critical distinctions to make when designing policies like the DGA. Moreover, pulses eaten as whole foods may promote gut health and mitigate obesity when they replace other more caloric foods or lipid-rich legumes, and pulse consumption has been associated with a myriad of health benefits, including healthy weight maintenance and a reduced risk for cardiovascular disease and several types of cancer [5,6]. 

## 4. The United States Dietary Guidelines as a Case in Point

In the recently released 2020–2025 DGA, what was termed the vegetable subgroup ‘legumes (beans and peas)’ in the previous DGA was changed to the ‘beans, peas, lentils’ vegetable subgroup [4]. This acknowledgement that beans, peas, and lentils, which the DGA recognizes as ‘pulses’ for the first time, have different nutritional properties than other legumes represents progress. However, the message becomes increasingly muddled: (1) edamame (an oilseed legume) is grouped with beans, peas, and lentils; (2) chickpeas are sometimes classified as peas (rather than a distinct type of pulse); (3) green beans (i.e., snap bean) are grouped with other vegetables; (4) green peas are separated and grouped with starchy vegetables; and (5) the beans, peas, and lentils subgroup is part of both vegetables and protein foods. Points one through four represent application of a confusing classification scheme that does not necessarily relay differences in nutritional composition. For instance, as is evident in Table 1, the nutritional profile of dry peas and green peas is strikingly similar per 100 kcal edible portion, yet the two are divided into different vegetable subgroups.

Arguably, as the guiding nutritional recommendations for the U.S., the DGA should achieve a level of clarity interpretable by health professionals as well as the consumer, yet the counterintuitive groupings of pulses and other legumes is likely confusing. Approximately two-thirds of Americans are overweight or obese, and clarity in dietary guidelines can play a critical role in improving the health of the nation. The DGA proclaim fiber a dietary component of public health concern, stating that, “More than 90 percent of women and 97 percent of men do not meet recommended intakes for dietary fiber” [4]. To mitigate this underconsumption and achieve adequate intake, eating patterns rich in fruits, vegetables, and whole grains have been heavily promoted. However, pulses are one of the richest, most economical sources of fiber, while simultaneously being low-fat and an excellent source of potassium, a second dietary component of public health concern. To readily increase fiber intake without exceeding caloric recommendations, the important role of pulses in the diet should be highlighted to both nutrition professionals and consumers. Yet, this requires clear dietary groupings and recommendations, which stem from appropriate use of terminology and classification.

One way to make comparisons between foods and design eating patterns that support reaching or maintaining a healthful weight is to compare 100 kcal portions. Using this approach, dry bean is compared to other foods in the vegetable, protein, and grain food groups in Table 2, Table 3 and Table 4, respectively. Dry bean was selected as a representative pulse because it accounts for the majority of pulse production worldwide [12]. Table 2 demonstrates that vegetables are naturally low in fat and are often high in fiber, depending on the subgroup (e.g., not starchy vegetables). However, pulses are nutritionally distinct from the other four vegetable subgroups (starchy, red and orange, dark-green, and other; see Table 2 caption) because they are a consistently rich source of protein and fiber, in about a 1-to-1 protein-to-fiber ratio. It is also important to consider serving size. A ½ cup of cooked pulses provides about 7 g of both protein and fiber. In contrast, it requires approximately 2 cups of cooked broccoli, which is already higher in protein and fiber than the other vegetables listed in the table, to obtain these levels of protein and fiber. Additionally, it requires 5 cups of raw cucumber to get about 4 g of protein and 3 g of fiber, which far exceeds the amounts eaten in a normal sitting yet is still not equivalent to higher levels seen in a serving size of pulses. Yet, other vegetable subgroups may have higher amounts of certain other dietary components than dry bean and other pulses. Whereas vitamin A and C are lacking in dry bean, they are present in high levels in vegetables such as carrots and broccoli, respectively.

Pulses are also dramatically different from animal protein foods (see Table 3), which contain no fiber. The animal proteins listed in Table 3 contain more protein per 100 kcal portion than pulses. However, fiber is a dietary component of public health concern, but protein is adequately consumed in the average American diet [4], reinforcing the need to make clear distinctions between foods and highlight those that are high in fiber. Other plant-based protein sources, such as nuts and soy products, contain fiber but have lower amounts than pulses and higher lipid content per 100 kcal portion. Indeed, although all protein sources shown in the table were prepared without added fat (e.g., oil), they are still naturally higher in lipid than pulses, again demonstrating the potential of pulses to meet dietary fiber recommendations within caloric limits. For example, 100 kcal of dry bean contains nearly 7 g of fiber, but almonds and tofu only contain about 2 to 3 g. The affordable price point of pulses can make them an accessible way for individuals from diverse socioeconomic classes to meet nutrition recommendations of dietary components such as fiber, protein, and potassium. Although the protein quality of plant-based foods is lower than animal sources due to limiting amino acids and lower bioavailability, healthy adults on a good quality diet that contains variety can achieve adequate protein intake even if animal proteins are excluded or minimally incorporated [13].

Overall, Table 2 and Table 3 demonstrate that pulses are distinct from other vegetable and protein foods. Pulses were also compared to cereal grains (Table 4), consumption of which has regularly been promoted as a strategy to increase consumption of dietary fiber. Pulses are consistently higher in protein than these grains, including quinoa which is often promoted as a source of protein. Importantly, pulses are also consistently about 2–3 times higher in fiber than whole grains [14,15]. For example, 100 kcal of dry bean has nearly 7 g of fiber, whereas brown rice and whole wheat pasta have approximately 1.3 and 2.6 g of fiber per 100 kcal, respectively. This difference is even more dramatic when comparing to a refined grain like white rice, with 100 kcal of white rice having only about 0.3 g of fiber. Pulses can also provide more of nutrients such as potassium and iron than grains.

## 5. Distinction among Legumes Moving Forward

Legumes can play a key role in healthful eating patterns around the world. Even beyond their central role in many plant-based diets, they can help meet nutritional needs in diets that include high amounts of animal protein by providing a rich source of dietary components like potassium and fiber. To best promote public health requires distinction among the various types of legumes and that a common vocabulary exist. Globally, there are discrepancies in the grouping and definition of legumes. While many countries consider them protein foods, some also group them as vegetables or even as a separate group [16]. For example, the United Kingdom Eatwell Guide groups undried legumes (e.g., runner beans and garden peas) with pulses and counts pulses as both protein and vegetables [17] but states that regardless of how much a consumer eats, “beans and pulses count as a maximum of 1 portion a day” of the vegetable group [18]. Conversely, Canada’s food guide groups beans, peas, and lentils with protein foods, with peas including chickpeas and dry peas [19].

Inconsistencies are also apparent in the 2020–2025 DGA. Although the DGA are “designed for policymakers and nutrition and health professionals to help all individuals and their families consume a healthy, nutritionally adequate diet” [4], we are witnessing rising rates of chronic disease and losing the battle against the obesity epidemic. The recently released DGA specify only four dietary components of public concern, attributing underconsumption to inadequate intake of nutrient-dense foods. Pulses are an excellent source of two of these dietary components, dietary fiber and potassium. Indeed, pulses seem like an obvious solution to eliminate the fiber gap, increase potassium intake, and contribute to the reversal of the trends of increasing obesity and chronic disease rates, all while not over-consuming calories. Moreover, they contain other health-promoting compounds, such as polyphenols with antioxidant properties [20].

The unclear grouping of pulses with various food groups and other legumes that have dramatically different nutritional profiles may contribute to the current health crisis by being a source of confusion to health care professionals, dietitians, and the public. In a recent study by Winham and colleagues (2018), Registered Dietitians (RDs) showed gaps in knowledge that reflect this conflicting messaging [21]. For example, 29% of the RDs did not know what ‘legume’ meant, and more than two-thirds were unable to define ‘pulse’. It would advantageous for the DGA to make distinctions among the different types of legumes, clearly highlighting the nutritional differences between oilseed legumes, undried legumes (e.g., snap beans or snap peas), and pulses (e.g., chickpeas, cowpeas, dry beans, dry peas, and lentils) such that nutrition and health care professionals can make appropriate recommendations to advance the health of their clients and the general public. With the current lack of clarity, incorporation of pulses into eating patterns across the lifespan may be diminished not because consumers are unwilling to eat pulses but because the DGA and health care professionals fail to give clear, specific guidance [22]. Distinction among different legumes and consistent use of terminology is essential to avoid public confusion, promote directed research (e.g., on food consumption patterns via pulse-specific food frequency questionnaires), and allow us to capitalize on the benefits that unique subgroups of legumes—such as pulses—have to offer.

## Figures and Tables

**Table 1:** Nutritional analysis of 100 kilocalorie portions of the predominant pulses, undried legumes, and oilseed legumes.

	Chickpea	Cowpea	Dry Bean	Snap Bean	Dry Pea	Green Pea	Snap Pea	Lentil	Peanut	Soybean	Edamame
**Approximate Amount**	~1/3 cup	~1/2 cup	~1/2–1/3 cup	~3 cups	~1/2 cup	~1 cup	~2.5 cups	~1/2 cup	~ 2 Tbs	~1/3 cup	~1/2 cup
**Protein (g)**	5.4	6.7	6.7	5.4	7.2	6.4	5.7	7.8	4.6	10.6	7.8
**Total Lipid (g)**	1.6	0.5	0.4	0.8	0.3	0.3	0.0	0.3	8.7	5.2	2.8
**Carbohydrate (g)**	16.7	17.9	18.0	22.5	17.7	18.6	17.2	17.4	2.8	4.9	11.1
**Dietary Fiber (g)**	4.6	5.6	6.6	9.1	7.2	6.5	5.9	6.8	1.5	3.5	3.4
**Folate (µg)**	104.9	179.3	112.9	94.3	56.0	75.0	MV	156.0	42.3	31.4	MV
**Iron (mg)**	1.8	2.2	1.6	1.9	1.1	1.8	5.7	2.9	0.8	3.0	1.9
**Potassium (mg)**	177.4	239.7	268.9	417.1	312.1	322.6	MV	318.1	124.3	299.4	365.6
**Calcium (mg)**	29.9	20.7	20.5	125.7	12.1	32.1	107.3	16.4	16.2	59.3	66.4
**Choline (mg)**	26.1	27.8	24.7	48.3	28.3	35.4	MV	28.2	9.3	MV	MV
**Magnesium (mg)**	29.3	45.7	53.0	51.4	31.0	46.4	MV	31.0	29.6	50.0	MV
**Vitamin A, RAE (µg)**	0.6	0.9	0.0	100.0	0.0	47.6	MV	0.0	0.0	0.0	MV
**Vitamin C (mg)**	0.8	0.3	0.0	27.7	0.3	16.9	MV	1.3	0.0	1.0	MV
**Vitamin E (mg)**	0.2	0.2	0.7	1.3	0.0	0.2	MV	0.1	1.5	0.2	MV
**FDC ID**	173757	175252	173735	169321	175257	170420	1130377	172421	1100536	174299	1132630

Information in the table is from the United States Department of Agriculture (USDA) FoodData Central (FDC), https://fdc.nal.usda.gov/ (accessed on 6 January 2021) [11]. The nutrition information for 100 kilocalorie portions are provided and FDC ID numbers are listed in the table. Dietary fiber, which USDA classifies as a dietary component of public health concern, is highlighted in green and lipid content is highlighted in blue. MV: Missing value in the database. All pulses and soybean were boiled and peanut is unroasted.

**Table 2:** Nutritional analysis of 100 kilocalorie portions of dry bean versus other vegetable subgroups.

	Dry Bean	Cassava	Potato	Carrots	Broccoli	Cucumber
**Approximate Amount**	~1/2–1/3 cup	~1/3 cup	~2/3 cup	~1.75 cups	~ 2 cups	~5 cups
**Protein (g)**	6.7	2.7	2.7	2.2	8.4	4.3
**Total Lipid (g)**	0.4	0.7	0.1	0.5	1.1	0.7
**Carbohydrate (g)**	18.0	22.6	22.7	23.5	19.7	24.2
**Dietary Fiber (g)**	6.6	0.7	2.4	8.6	7.7	3.3
**Folate (µg)**	112.9	MV	30.1	40.0	160.0	46.7
**Iron (mg)**	1.6	0.2	1.2	1.0	2.2	1.9
**Potassium (mg)**	268.9	252.7	573.1	671.4	937.1	980.0
**Calcium (mg)**	20.5	13.0	16.1	85.7	140.0	106.7
**Choline (mg)**	24.7	MV	15.8	25.1	55.4	40.0
**Magnesium (mg)**	53.0	MV	30.1	28.6	62.9	86.7
**Vitamin A, RAE (µg)**	0.0	MV	1.1	2434.3	88.6	33.3
**Vitamin C (mg)**	0.0	16.0	10.3	10.3	224.9	18.7
**Vitamin E (mg)**	0.7	MV	0.0	2.9	2.3	0.2
**FDC ID**	173735	372909	1102892	170394	1103172	1103352

All five vegetable subgroups in the DGA are represented: (1) beans, peas, lentils by cooked dry bean; (2) starchy by cas-sava and potatoes; (3) red & orange by carrots; (4) dark-green by broccoli; and (5) other by cucumber. Dietary fiber, which USDA classifies as a dietary component of public health concern, is highlighted in green and lipid content is highlighted in blue. MV: Missing value in the database. To make for valid comparisons, all vegetable selections are prepared without added fat (e.g., oil) and are cooked, except for cucumber which is shown raw to represent how it is normally eaten. Information from USDA FDC [11].

**Table 3:** Nutritional analysis of 100 kilocalorie portions of dry bean versus other protein foods.

	Dry Bean	Chicken Breast, Skin not Eaten	80/20 Ground Beef	Hard-Boiled Egg	Salmon	Almonds, Unroasted	Tofu
**Approximate Amount**	~1/2–1/3 cup	~2–oz.	~1.3–oz.	~1.25 eggs	~2–oz.	~2 Tbs	~3.5–oz.
**Protein (g)**	6.7	16.8	8.1	8.1	16.1	3.7	10.0
**Total Lipid (g)**	0.4	3.1	5.6	6.8	3.5	8.6	5.6
**Carbohydrate (g)**	18.0	0.0	0.0	0.7	0.1	3.7	2.5
**Dietary Fiber (g)**	6.6	0.0	0.0	0.0	0.0	2.2	2.6
**Folate (µg)**	112.9	4.0	3.1	28.4	3.1	7.6	MV
**Iron (mg)**	1.6	0.3	0.8	0.8	0.3	0.6	1.8
**Potassium (mg)**	268.9	200.0	95.6	81.3	288.8	126.6	156.4
**Calcium (mg)**	20.5	4.0	7.6	32.3	5.6	46.5	187.2
**Choline (mg)**	24.7	43.0	25.4	189.7	74.5	9.0	MV
**Magnesium (mg)**	53.0	15.9	6.3	6.5	21.3	46.6	MV
**Vitamin A, RAE (µg)**	0.0	5.1	0.9	96.1	25.0	0.0	0.0
**Vitamin C (mg)**	0.0	0.0	0.0	0.0	0.3	0.0	0.0
**Vitamin E (mg)**	0.7	0.6	0.0	0.7	0.3	4.4	MV
**FDC ID**	173735	1098457	171797	173424	1098965	1100508	1219633

All subgroups of protein foods in the DGA are represented: (1) beans, peas, lentils by cooked dry bean; (2) meats, poultry, eggs by chicken breast, ground beef, and hard-boiled eggs; (3) seafood by salmon; and (4) nuts, seeds, soy products by almonds and tofu. Dietary fiber, which USDA classifies as a dietary component of public health concern, is highlighted in green and lipid content is highlighted in blue. MV: Missing value in the database. No fat (e.g., oil) was added when preparing these foods. Information from USDA FDC [11].

**Table 4:** Nutritional analysis of 100 kilocalorie portions of dry bean versus common grains.

	Dry Bean	Rice, Brown	Rice, White	Whole Wheat Pasta	Corn Tortilla	Corn Grits	Quinoa
**Approximate Amount**	~1/2–1/3 cup	~0.4 cup	~1/2 cup	~2/3 cup	~2 small tortillas	~2/3 cup	~1/2 cup
**Protein (g)**	6.7	2.2	2.1	4.0	2.2	1.9	3.7
**Total Lipid (g)**	0.4	0.8	0.2	1.1	1.1	0.6	1.6
**Carbohydrate (g)**	18.0	20.0	21.7	20.2	20.0	21.4	17.8
**Dietary Fiber (g)**	6.6	1.3	0.3	2.6	2.2	1.1	2.3
**Folate (µg)**	112.9	7.4	45.0	14.1	MV	41.5	35.0
**Iron (mg)**	1.6	0.5	0.9	1.2	0.8	0.9	1.2
**Potassium (mg)**	268.9	70.5	27.1	64.4	MV	33.8	143.3
**Calcium (mg)**	20.5	2.5	7.8	8.7	89.0	1.5	14.2
**Choline (mg)**	24.7	7.5	1.6	4.4	MV	3.7	19.2
**Magnesium (mg)**	53.0	32.0	9.3	36.2	MV	7.7	53.3
**Vitamin A, RAE (µg)**	0.0	0.0	0.0	0.0	MV	0.0	0.0
**Vitamin C (mg)**	0.0	0.0	0.0	0.0	0.0	0.0	0.0
**Vitamin E (mg)**	0.7	0.1	0.0	0.2	MV	0.0	0.5
**FDC ID**	173735	1101631	1101625	168910	468846	171672	168917

Values are from cooked dry bean and grains, with no fat (e.g., oil) added during preparation. Dietary fiber, which USDA classifies as a dietary component of public health concern, is highlighted in green and lipid content is highlighted in blue. MV: Missing value in the database. Information from USDA FDC [11].

## Data Availability

Publicly available datasets were analyzed for the tables presented in this communication. This data can be found at the USDA FDC: https://fdc.nal.usda.gov/ (accessed on 6 January 2021).
